# Supplementary material for: Differentiating iron-loading anemias using a newly developed and analytically validated ELISA for human serum erythroferrone
Source: PLoS One. 2021 Jul 20;16(7):e0254851. doi: 10.1371/journal.pone.0254851 (PMC8291690; doi:10.1371/journal.pone.0254851)
Supplement: S1 Methods — (DOCX) [file pone.0254851.s001.docx]

**Supplemental methods**

*Polyclonal anti-hERFE antibodies*

Both chickens and rabbits were injected 4 or 5 times bimonthly, followed by collection of either eggs or whole blood, respectively. Isolation of the IgY fraction of the egg yolks, preparation of the antisera and all related animal experiments were performed by Davids Biotechnology (Regensburg, Germany) following standard protocol [1]. All polyclonal antibodies were purified by affinity chromatography (AffiGel®15, Bio-Rad Laboratories, Hercules, CA, USA) followed by ultrafiltration, as described previously [2]. The purified antibodies of both chickens as well of those of both rabbits were pooled, diluted with 50% glycerol and stored in aliquots at -20 ^0^C.

Laboratory measurements

For all patient and healthy control samples C-reactive protein (CRP), EPO, hemoglobin (Hb), mean corpuscular volume (MCV) of red blood cells and serum iron parameters were measured in ISO-15189 accredited Dutch hospital laboratories. Serum hepcidin-25 measurements were performed by a standardized Weak Cation Exchange Time-Of-Flight mass spectrometry (WCX-TOF MS) method [3, 4]. Additionally, ERFE concentration was also measured using a commercial ERFE kit from Intrinsic Life Science [5] and a previously described in-house ELISA [6] to compare the performance of the new assay.

*Sample collection and processing*

Samples of the biobank are derived from blood collections before either blood transfusion or phlebotomy between December 2016 and August 2019. After 1-2 hours of coagulation, samples were centrifuged at 2000g for 10 minutes and the serum samples were stored in liquid nitrogen within 4 hours of sampling. Upon issuance, the samples were thawed, aliquoted and stored at -80 ^0^C until measurement in August 2020. Control samples from volunteers were collected in June 2018, following the same collection protocol as described for the biobank samples. After centrifugation, serum samples were aliquoted and stored at -80 ^0^C within 4 hours of sampling until measurement in August 2020. Volunteers who used any sort of medication, except birth control pills, or who had been previously diagnosed with any iron disorder were excluded.

*ELISA procedure*

ELISA plates were coated overnight at 4 °C with chicken anti-hERFE as the capture antibodies in coating buffer (1.2 µg/mL). After washing, the plates were blocked with 300 µL dilution buffer for 2 hours and washed again. The hERFE standard (10 ng/µL) was serial diluted between 4 and 0.125 ng/mL and incubated overnight at 4°C together with the diluted serum samples, all in duplicate. Next, after washing, both the rabbit anti-hERFE tagging antibodies in dilution buffer (0.6 µg/mL), or solely dilution buffer to produce a nonsense signal, were incubated for 1 hour at room temperature (RT) under moderate mixing. Plates were then washed, followed by a 1 hour incubation at RT under moderate mixing with anti-rabbit IgG-HRP antibody and subsequent washing. All washing steps consisted of 4 times 300 µL of washing buffer per well. The color reaction was started with addition of substrate solution TMB One and stopped with 0.2M H_2_SO_4_ after 20 minutes, after which OD was measured at 450 nm. An acceptance limit of 25% for a duplicate CV is defined.

*ELISA format*

A so-called ‘nonsense format’ is used to quantify potential interference of heterophilic antibodies present in sample matrix [7]. Each sample of interest was measured in duplicate following the procedure described above, thereby obtaining the sense signal. By measuring each sample in duplicate without the tagging antibody, a nonsense signal was obtained. Next, the mean OD of the dilution buffer was subtracted from the nonsense signal to quantify the aspecific signal. If greater than zero, the aspecific signal was subtracted from the sense signal to obtain a corrected OD value. If the aspecific signal was not found greater than zero, the sense signal was used for further calculations.

## *Equipment and reagents*

MICROLON®600 flat bottomed 96-wells ELISA plates (Greiner Bio-one, Kremsmünster, Austria, #655092) were used. Washing procedures were performed using a Hydroflex plate washer (Tecan, Männedorf, Switzerland). Optical density (OD) values were measured using a Tecan Infinite® F50 ELISA reader with Magellan software (Tecan, version 7.0).

Goat anti-Rabbit IgG peroxidase antibody (#A0545) and bovine serum albumin (BSA, #A7030) were purchased from Sigma Aldrich (St. Louis, MO, USA). Pierce peroxidase conjugate stabilizer (#31503) was supplied from Thermo Scientific (Waltham, MA, United States) and TMB One (#4380) from Kementec (Taastrup, Denmark). All other reagents used were of analytical grade.

MiliQ water was used for the following buffers: washing buffer (PBS, 0.1% Tween-20), dilution buffer (washing buffer, 1% BSA), coating buffer (15 mM Na_2_CO_3_, 35 mM NaHCO_3_, pH 9.6) and stop solution (0.2M H_2_SO_4_).

Recombinant Flag-tagged human ERFE (hERFE) was produced and purified as previously described [6] and used for immunization, affinity purification and as assay standard.

**References**

1. Available at:<https://www.davids-bio.com/pages/protocols.html>. Accessed: 4 May 2021.

2. Grebenschikov N, Geurts-Moespot A, De Witte H, Heuvel J, Leake R, Sweep F, et al. A sensitive and robust assay for urokinase and tissue-type plasminogen activators (upa and tpa) and their inhibitor type i (pai-1) in breast tumor cytosols. Int J Biol Markers. 1997;12:6-14.

3. Laarakkers CMM, Wiegerinck ET, Klaver S, Kolodziejczyk M, Gille H, Hohlbaum AM, et al. Improved mass spectrometry assay for plasma hepcidin: Detection and characterization of a novel hepcidin isoform. PLOS ONE. 2013;8:e75518.

4. Diepeveen LE, Laarakkers CMM, Martos G, Pawlak ME, Uğuz FF, Verberne K, et al. Provisional standardization of hepcidin assays: Creating a traceability chain with a primary reference material, candidate reference method and a commutable secondary reference material. Clin Chem Lab Med. 2019;57:864-72.

5. Han H, Westerman K, Ostland V, Gutschow P, Olbina G, da Silva Guimarães J, et al. A novel dual monoclonal sandwich elisa for human erythroferrone. Blood. 2016;128:1272-.

6. Ganz T, Jung G, Naeim A, Ginzburg Y, Pakbaz Z, Walter PB, et al. Immunoassay for human serum erythroferrone. Blood. 2017;130:1243-6.

7. Grebenchtchikov N, Sweep CG, Geurts-Moespot A, Piffanelli A, Foekens JA, Benraad TJ. An elisa avoiding interference by heterophilic antibodies in the measurement of components of the plasminogen activation system in blood. J Immunol Methods. 2002;268:219-31.
