# Supplementary material for: Differentiating iron-loading anemias using a newly developed and analytically validated ELISA for human serum erythroferrone
Source: PLoS One. 2021 Jul 20;16(7):e0254851. doi: 10.1371/journal.pone.0254851 (PMC8291690; doi:10.1371/journal.pone.0254851)
Supplement: S1 Table — Nine patient samples with high endogenous ERFE concentrations were serially diluted. Recovery was calculated using the ERFE concentration determined at dilution 1:5, 1:10 or 1:20 as a reference value. Mean recovery of the subsequent dilution (1:10, 1:15 or 1:40) was 117% (range 44–211%), 133% (range 50–305%) or 119% (range 29–295%), respectively. << represent ERFE concentrations below LLOQ. (DOCX) [file pone.0254851.s002.docx]

|  | Sample 1 | | Sample 2 | | Sample 3 | | Sample 4 | | Sample 5 | | Sample 6 | | Sample 7 | | Sample 8 | | Sample 9 | |
| --- | --- | --- | --- | --- | --- | --- | --- | --- | --- | --- | --- | --- | --- | --- | --- | --- | --- | --- |
| Dilution factor | Conc.  (ng/mL) | Recov. (%) | Conc.  (ng/mL) | Recov. (%) | Conc.  (ng/mL) | Recov. (%) | Conc.  (ng/mL) | Recov. (%) | Conc.  (ng/mL) | Recov. (%) | Conc.  (ng/mL) | Recov. (%) | Conc.  (ng/mL) | Recov. (%) | Conc.  (ng/mL) | Recov. (%) | Conc.  (ng/mL) | Recov. (%) |
| **5** | **2.59** | **100** | **2.45** | **100** | **1.10** | **100** | **4.64** | **100** | **3.62** | **100** | **2.64** | **100** | **3.67** | **100** | **1.22** | **100** | **3.28** | **100** |
| 10 | 1.93 | 75 | 2.93 | 120 | 2.32 | 211 | 5.40 | 116 | 1.59 | 44 | 3.84 | 145 | 4.23 | 115 | 1.43 | 117 | 3.71 | 113 |
| 15 | 2.05 | 79 | 2.53 | 103 | 1.46 | 133 | 6.20 | 134 | 4.84 | 134 | 3.60 | 136 | 7.29 | 199 | 2.97 | 244 | 1.86 | 57 |
| 20 | 2.20 | 85 | 1.88 | 77 | 1.37 | 125 | 8.78 | 189 | 5.51 | 152 | 5.84 | 221 | 8.06 | 220 | 3.32 | 273 | 2.28 | 70 |
| 40 | 3.47 | 134 | 3.48 | 142 | 4.03 | 367 | 8.18 | 176 | 1.82 | 50 | 4.84 | 183 | 6.04 | 165 | << |  | 0.66 | 20 |

|  | Sample 1 | | Sample 2 | | Sample 3 | | Sample 4 | | Sample 5 | | Sample 6 | | Sample 7 | | Sample 8 | | Sample 9 | |
| --- | --- | --- | --- | --- | --- | --- | --- | --- | --- | --- | --- | --- | --- | --- | --- | --- | --- | --- |
| Dilution factor | Conc.  (ng/mL) | Recov. (%) | Conc.  (ng/mL) | Recov. (%) | Conc.  (ng/mL) | Recov. (%) | Conc.  (ng/mL) | Recov. (%) | Conc.  (ng/mL) | Recov. (%) | Conc.  (ng/mL) | Recov. (%) | Conc.  (ng/mL) | Recov. (%) | Conc.  (ng/mL) | Recov. (%) | Conc.  (ng/mL) | Recov. (%) |
| 5 | 2.59 | 134 | 2.45 | 84 | 1.10 | 47 | 4.64 | 86 | 3.62 | 228 | 2.64 | 69 | 3.67 | 87 | 1.22 | 85 | 3.28 | 88 |
| **10** | **1.93** | **100** | **2.93** | **100** | **2.32** | **100** | **5.40** | **100** | **1.59** | **100** | **3.84** | **100** | **4.23** | **100** | **1.43** | **100** | **3.71** | **100** |
| 15 | 2.05 | 106 | 2.53 | 86 | 1.46 | 63 | 6.20 | 115 | 4.84 | 305 | 3.60 | 94 | 7.29 | 172 | 2.97 | 208 | 1.86 | 50 |
| 20 | 2.20 | 114 | 1.88 | 64 | 1.37 | 59 | 8.78 | 163 | 5.51 | 347 | 5.84 | 152 | 8.06 | 191 | 3.32 | 233 | 2.28 | 62 |
| 40 | 3.47 | 180 | 3.48 | 119 | 4.03 | 174 | 8.18 | 152 | 1.82 | 114 | 4.84 | 126 | 6.04 | 143 | << |  | 0.66 | 18 |

|  | Sample 1 | | Sample 2 | | Sample 3 | | Sample 4 | | Sample 5 | | Sample 6 | | Sample 7 | | Sample 8 | | Sample 9 | |
| --- | --- | --- | --- | --- | --- | --- | --- | --- | --- | --- | --- | --- | --- | --- | --- | --- | --- | --- |
| Dilution factor | Conc.  (ng/mL) | Recov. (%) | Conc.  (ng/mL) | Recov. (%) | Conc.  (ng/mL) | Recov. (%) | Conc.  (ng/mL) | Recov. (%) | Conc.  (ng/mL) | Recov. (%) | Conc.  (ng/mL) | Recov. (%) | Conc.  (ng/mL) | Recov. (%) | Conc.  (ng/mL) | Recov. (%) | Conc.  (ng/mL) | Recov. (%) |
| 5 | 2.59 | 118 | 2.45 | 130 | 1.10 | 80 | 4.64 | 53 | 3.62 | 66 | 2.64 | 45 | 3.67 | 45 | 1.22 | 37 | 3.28 | 144 |
| 10 | 1.93 | 88 | 2.93 | 155 | 2.32 | 170 | 5.40 | 62 | 1.59 | 29 | 3.84 | 66 | 4.23 | 52 | 1.43 | 43 | 3.71 | 162 |
| 15 | 2.05 | 93 | 2.53 | 134 | 1.46 | 107 | 6.20 | 71 | 4.84 | 88 | 3.60 | 62 | 7.29 | 90 | 2.97 | 90 | 1.86 | 81 |
| **20** | **2.20** | **100** | **1.88** | **100** | **1.37** | **100** | **8.78** | **100** | **5.51** | **100** | **5.84** | **100** | **8.06** | **100** | **3.32** | **100** | **2.28** | **100** |
| 40 | 3.47 | 158 | 3.48 | 185 | 4.03 | 295 | 8.18 | 93 | 1.82 | 33 | 4.84 | 83 | 6.04 | 75 | << |  | 0.66 | 29 |
