# Supplementary material for: Differentiating iron-loading anemias using a newly developed and analytically validated ELISA for human serum erythroferrone
Source: PLoS One. 2021 Jul 20;16(7):e0254851. doi: 10.1371/journal.pone.0254851 (PMC8291690; doi:10.1371/journal.pone.0254851)
Supplement: S2 Table — * Treatment at the time of sample collection. ** g/L = 16.1 mM. *** Standardized hepcidin value. (DOCX) [file pone.0254851.s003.docx]

| **ID** | **Gender**  **M/F** | **Age**  **yrs** | **Diagnosis** | **Treatment*** | **Hb (mM)**** | **MCV (fl)** | **Iron (µM)** | **TIBC (µM)** | **TSAT (%)** | **Ferritin (µg/L)** | **CRP (mg/L)** | **EPO (U/L)** | **Hepcidin (nM)***** | **Hepcidin/ferritin ratio (pmol/µg)** | **ERFE (ng/mL)** |
| --- | --- | --- | --- | --- | --- | --- | --- | --- | --- | --- | --- | --- | --- | --- | --- |
| P1 | M | 24 | β-thal M | Transfusion and chel. | 5.8 | 80 | 36 | 46 | 78 | 563 | <1 | 48.5 | 0.5 | 0.89 | 3.95 |
| P2 | F | 27 | β-thal M | Transfusion and chel. | 5.2 | 82 | 32 | 33 | 97 | 833 | <1 | 39.8 | 1.3 | 1.56 | 6.26 |
| P3 | F | 32 | β-thal M | Transfusion and chel. | 5.7 | 83 | 45 | 43 | 105 | 1358 | 1 | 191 | 7.5 | 5.52 | 3.08 |
| P4 | M | 30 | β-thal M | Transfusion and chel. | 4.8 | 77 | 35 | 38 | 92 | 721 | 1 | 129 | 2.0 | 2.77 | 4.55 |
| P5 | M | 47 | β-thal M | Transfusion and chel. | 5.3 | 78 | 46 | 49 | 94 | 564 | <1 | 48.7 | <0.5 | 0.44 | 4.86 |
| P6 | F | 49 | β-thal M | Transfusion and chel. | 5.7 | 67 | 26 | 32 | 81 | 576 | <1 | 199 | 3.9 | 6.77 | 2.70 |
| P7 | M | 20 | β-thal M | Transfusion and chel. | ND | ND | 42 | 43 | 98 | 5376 | <1 | 56.3 | 6.1 | 1.13 | 3.38 |
| P8 | M | 37 | XLSA | Phlebotomy | 7.7 | 73 | 34 | 61 | 56 | 76 | <1 | 10.5 | 2.0 | 26.32 | 1.91 |
| P9 | M | 16 | XLSA | None | 6.8 | 69 | 28 | 66 | 42 | 58 | <1 | 22.8 | 3.3 | 56.90 | 2.70 |
| P10 | M | 72 | XLSA | Phlebotomy and chel. | 7.7 | 75 | 28 | 58 | 48 | 226 | <1 | 7.4 | 4.3 | 19.03 | 2.13 |
| P11 | M | 24 | XLSA | Phlebotomy | 7.1 | 64 | 38 | 43 | 88 | 382 | 1 | 9.1 | <0.5 | 0.65 | 3.27 |
| P12 | M | 58 | XLSA | Phlebotomy | 8.0 | 75 | 26 | 65 | 40 | 148 | <1 | 17.6 | 1.2 | 8.11 | 3.57 |
| P13 | M | 20 | XLSA | None | ND | ND | 22 | 54 | 41 | 301 | 2 | 9.9 | 3.8 | 12.62 | 1.74 |
| P14 | M | 40 | XLSA | None | 7.8 | 74 | 19 | 48 | 40 | 573 | ND | ND | 8.9 | 15.53 | 1.91 |
| C1 | M | 53 | N/A | | ND | | 19 | 68 | 28 | 245 | <1 | 8.8 | 6.4 | 26.12 | 1.18 |
| C2 | F | 25 |  |  |  |  | 14 | 69 | 20 | 33 | <1 | 7.2 | 0.5 | 15.15 | <1.16 |
| C3 | F | 46 |  |  |  |  | 5 | 76 | 7 | 11 | <1 | 13.5 | <0.5 | 22.73 | 1.69 |
| C4 | M | 26 |  |  |  |  | 26 | 50 | 52 | 86 | <1 | 4.2 | 1.4 | 16.28 | <1.16 |
| C5 | M | 27 |  |  |  |  | 11 | 76 | 14 | 112 | 1 | 15.3 | 2.1 | 18.75 | <1.16 |
| C6 | M | 52 |  |  |  |  | 25 | 62 | 40 | 385 | <1 | 9.7 | 12.1 | 31.43 | 1.76 |
| C7 | F | 55 |  |  |  |  | 18 | 76 | 24 | 14 | 1 | 22.6 | 0.7 | 50.00 | 1.29 |
| C8 | M | 33 |  |  |  |  | 34 | 72 | 47 | 130 | <1 | 7.1 | 2.3 | 17.69 | 1.88 |
| C9 | F | 26 |  |  |  |  | 26 | 82 | 32 | 97 | 1 | 11.1 | 3.9 | 40.21 | 1.75 |
| C10 | F | 39 |  |  |  |  | 9 | 66 | 14 | 17 | <1 | 14.1 | <0.5 | 14.71 | 2.01 |
| C11 | M | 56 |  |  |  |  | 22 | 50 | 44 | 160 | <1 | 6.0 | 3.1 | 19.38 | 1.44 |
| C12 | F | 20 |  |  |  |  | 22 | 72 | 31 | 36 | 2 | 8.0 | 2.0 | 55.56 | 1.37 |
| C13 | M | 36 |  |  |  |  | 7 | 57 | 12 | 205 | 1 | 7.1 | 10.9 | 53.17 | <1.16 |
| C14 | F | 27 |  |  |  |  | 19 | 62 | 31 | 18 | <1 | 7.9 | <0.5 | 13.89 | 1.87 |
| C15 | F | 20 |  |  |  |  | 22 | 92 | 24 | 21 | 1 | 6.9 | <0.5 | 11.90 | <1.16 |
